# Insulin micro-secretion in Type 1 diabetes and related microRNA profiles

**DOI:** 10.1038/s41598-021-90856-6

**Published:** 2021-06-03

**Authors:** Andrzej S. Januszewski, Yoon Hi Cho, Mugdha V. Joglekar, Ryan J. Farr, Emma S. Scott, Wilson K. M. Wong, Luke M. Carroll, Yik W. Loh, Paul Z. Benitez-Aguirre, Anthony C. Keech, David N. O’Neal, Maria E. Craig, Anandwardhan A. Hardikar, Kim C. Donaghue, Alicia J. Jenkins

**Affiliations:** 1 NHMRC Clinical Trials Centre, University of Sydney, Sydney, NSW Australia; 2 Department of Medicine, University of Melbourne, Melbourne, VIC Australia; 3 Discipline of Paediatrics and Child Health, University of Sydney, Sydney, NSW Australia; 4 Institute of Endocrinology and Diabetes, The Children’s Hospital at Westmead, Sydney, NSW Australia; 5 School of Medicine, Western Sydney University, Sydney, NSW Australia; 6 Department of Science and Environment, Roskilde University, Copenhagen, Denmark

**Keywords:** Type 1 diabetes, Genetic markers

## Abstract

The aim of this cross-sectional study was to compare plasma C-peptide presence and levels in people without diabetes (CON) and with Type 1 diabetes and relate C-peptide status to clinical factors. In a subset we evaluated 50 microRNAs (miRs) previously implicated in beta-cell death and associations with clinical status and C-peptide levels. Diabetes age of onset was stratified as adult (≥ 18 y.o) or childhood (< 18 y.o.), and diabetes duration was stratified as ≤ 10 years, 10–20 years and > 20 years. Plasma C-peptide was measured by ultrasensitive ELISA. Plasma miRs were quantified using TaqMan probe-primer mix on an OpenArray platform. C-peptide was detectable in 55.3% of (n = 349) people with diabetes, including 64.1% of adults and 34.0% of youth with diabetes, *p* < 0.0001 and in all (n = 253) participants without diabetes (CON). C-peptide levels, when detectable, were lower in the individuals with diabetes than in the CON group [median lower quartile (LQ)–upper quartile (UQ)] 5.0 (2.6–28.7) versus 650.9 (401.2–732.4) pmol/L respectively, *p* < 0.0001 and lower in childhood versus adult-onset diabetes [median (LQ–UQ) 4.2 (2.6–12.2) pmol/L vs. 8.0 (2.3–80.5) pmol/L, *p* = 0.02, respectively]. In the childhood-onset group more people with longer diabetes duration (> 20 years) had detectable C-peptide (60%) than in those with shorter diabetes duration (39%, *p* for trend < 0.05). Nine miRs significantly correlated with detectable C-peptide levels in people with diabetes and 16 miRs correlated with C-peptide levels in CON. Our cross-sectional study results are supportive of (a) greater beta-cell function loss in younger onset Type 1 diabetes; (b) persistent insulin secretion in adult-onset diabetes and possibly regenerative secretion in childhood-onset long diabetes duration; and (c) relationships of C-peptide levels with circulating miRs. Confirmatory clinical studies and related basic science studies are merited.

## Introduction

Type 1 diabetes arises from the autoimmune-induced loss of insulin producing pancreatic cells, with ≈70% loss at clinical presentation^1^. However, even people with long-term diabetes can have low level insulin production^2,3^, which is associated with better glycaemia^4^ and reduced risk of severe hypoglycaemia (SH)^5,6^ and chronic complications^6,7^. Recently, ultrasensitive C-peptide assays have demonstrated that the majority of people with diabetes have residual C-peptide “micro-secretion”, suggesting that some beta cells have escaped immune attack or regenerated^8^. The lower limit of detection of routine clinical laboratory C-peptide assays is ≈50 pmol/L, while that of ultrasensitive research assays is 1.5–2.5 pmol/L, facilitating detailed examination of residual beta cell function and its relationship to age of diabetes onset and duration^8–12^, as a biomarker for clinical outcomes^12^ and in clinical trials of interventions to retard Type 1 diabetes.

MicroRNAs (miRs) are small (18–22 nucleotide) non-coding (nc)RNAs that post-transcriptionally regulate gene expression by targeted inhibition or degradation of messenger (m)RNA. They are stable in plasma/serum, resistant to multiple freeze–thaw cycles and pH-mediated degradation and relatively easy to detect using quantitative (q)PCR. Various miRs have shown different expression in relation to Type 1 and Type 2 diabetes development and/or progression^13^.

We measured C-peptide levels in a cross-sectional study of paediatric and adult participants with Type 1 diabetes and people without diabetes (controls, CON). We compared plasma C-peptide absence or presence (as a categorical variable) and related C-peptide levels (as a continuous variable) to diabetes age of onset (childhood or adult), diabetes duration and HbA1c levels in study participants with diabetes and detectable C-peptide. In the paediatric diabetes group, we also examined the relationships between the presence of autoantibodies and C-peptide. Furthermore, in a subset of adult and paediatric diabetic and CON participants we evaluated 50 miRs previously implicated in beta cell death^14^ in relationship to diabetes status, age of diabetes onset and duration and C-peptide levels as a qualitative and quantitative factor.

## Results

### Participant characteristics

Participant characteristics are summarised in Table 1, including 253 CON and 349 participants with diabetes and known complication status, aged 10–80 years and median (LQ–UQ) diabetes duration of 12 (7–22) years. As the diabetes complication status was unknown in 11 participants their results are not included in Table 1.

**Table 1:** Clinical characteristics of participants with and without diabetes.

	Controls	Type 1 diabetes CX−	Type 1 diabetes CX+
n (% men)	253 (44.7)	221 (44.3)	117 (47.0)
Age (years)	36 ± 15	30 ± 15*	33 ± 18
T1D duration (years)	–	14 ± 11	21 ± 14†
HbA1c (%)	5.1 ± 0.4	8.0 ± 1.2*	8.9 ± 2.0*†
HbA1c (mmol/mol)	32.4 ± 4.1	64.3 ± 13.1*	73.7 ± 21.9*†
At HbA1c target (%)^a^	–	21.9	17.7
Total Cholesterol (mmol/L)	5.2 ± 1.0	4.6 ± 0.9*	4.8 ± 1.2*
HDL–C (mmol/L)	1.5 ± 0.4	1.5 ± 0.4	1.4 ± 0.4
LDL-C (mmol/L)	3.1 ± 1.0	2.7 ± 0.9*	2.8 ± 1.0
BMI (kg/m2)	25.4 ± 4.3	24.7 ± 4.0	26.6 ± 6.3†
SBP (mmHg)	121 ± 15	117 ± 15*	126 ± 21†
DBP (mm Hg)	69 ± 10	66 ± 9*	69 ± 12†
Pulse Pressure (mmHg)	52 ± 9	54 ± 9	63 ± 13*†
Serum creatinine (µmol/L)	80 (70, 90)	80 (70, 90)	90 (80, 107)*†
Urine ACR (mg/mmol)	0.47 (0.32, 0.80)	0.50 (0.35, 0.87)	2.21 (0.89, 15.51)*†
eGFR (ml/min/m^2^)	104 (88, 119)	108 (89, 134)	96 (72, 120)†
Retinopathy n (%)	–	–	79 (68)
Nephropathy n (%)	–	–	82 (71)
CVD n (%)	–	–	20 (17)

Data shown: mean ± SD or median (LQ, UQ).

*p < 0.05 versus CON.

^†^*p* < 0.05 versus Type 1 diabetes CX-.

^a^HbA1c target: ≤ 7% (53 mmol/mol) for adults, ≤ 7.5% (58 mmol/mol) for adolescents.

### C-peptide correlates with age of diabetes diagnosis and duration

Plasma was derived from blood taken from fasted adults and from children with variable prandial status. C-peptide was detectable in all CON subjects and in 55.3% (193 of 349) of participants with diabetes (63.8% (159 of 249) of adults and 34.0% (34 of 100) of youth, *p* < 0.0001).

**Figure 1:**
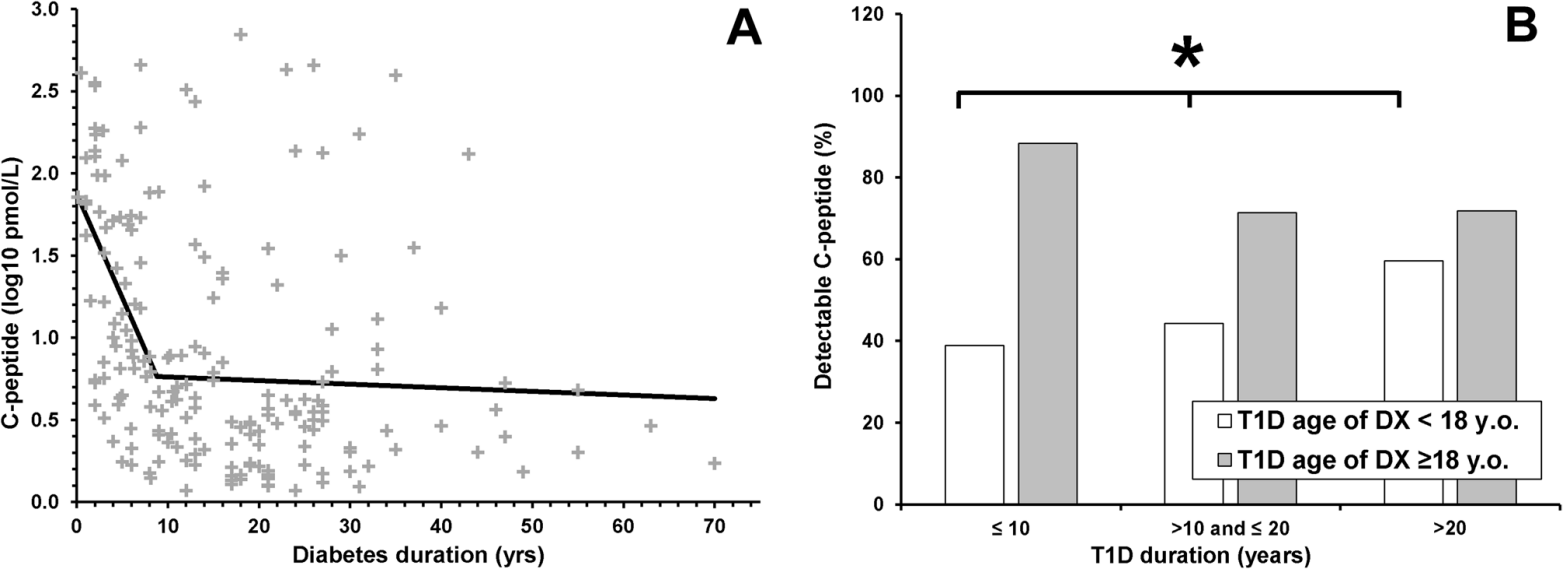
(**A**) Segmental linear regression of detectable C-peptide levels versus Type 1 diabetes duration. Overall *r* = 0.45, slope before threshold *p* = 0.0005, slope after threshold *p* = 0.66; diabetes duration threshold 8.8 (95% CI 5.7, 11.9) years (*p* < 0.0001). (**B**) Comparison of proportion of detectable C-peptide in participants with diabetes stratified by age of diabetes diagnosis and duration. Significant positive trend in percentage of detectable C-peptide with diabetes duration in diabetes diagnosed below 18 y.o. (*p* = 0.01) and borderline negative trend in diabetes diagnosed ≥ 18 y.o. (*p* = 0.07).

In the participants with diabetes with detectable C-peptide median (LQ–UQ)) C-peptide levels were significantly lower than in the CON group 5.0 (2.6–28.7) pmol/L versus 650.9 (401.2–732.4) pmol/L respectively, *p* < 0.0001. In participants with diabetes and detectable C-peptide, C-peptide levels correlated (segmental regression) with age of diabetes diagnosis (*r* = 0.23; *p* = 0.001) and inversely with diabetes duration *r* = −0.45; *p* = 0.003. The latter was non-linear, with a steeper slope at shorter diabetes duration. In segmental regression analyses of C-peptide levels versus diabetes duration, the *p*-values of the slopes before and after the duration cut-point of 8.8 (95% CI 5.7, 11.9) years (*p* < 0.0001) were 0.0005 and 0.66 respectively (Fig. 1A).

### C-peptide and autoantibody status

In 100 youth with diabetes (median age at assessment 15 y.o. and 8 years diabetes duration) IA2 and GAD status was known in 85 participants: (60 (70.5%) were positive for ≥ 1 auto antibody), 47% were GAD positive, of whom 39% had detectable C-peptide, while 44% were IA2 positive, of whom 40% had detectable C-peptide. Overall, in those positive for ≥ 1 antibody 38.8% had detectable C-peptide. There was no difference in C-peptide levels by antibody status (GAD + vs -; *p* = 0.22 and IA2 + vs -; *p* = 0.37). The only clinical parameter different between those with versus without autoantibodies was SBP: 111 ± 12 versus 101 ± 13 mmHg respectively; *p* = 0.0003.

### Different detectable C-peptide rates and levels by age of diabetes onset and duration

Plasma C-peptide was detectable in 46.3% (114 of 246) and 79.2% (76 of 96) of participants with diabetes aged < 18 y.o. and ≥ 18 y.o. at diabetes diagnosis respectively, *p* < 0.0001. In those with detectable C-peptide, the median C-peptide levels were lower in participants with diabetes with earlier (< 18 y.o.) versus later onset (≥ 18 y.o.) diabetes: 4.2 (2.6–12.2) pmol/L versus 8.0 (2.3–80.5) pmol/L, *p* = 0.02. C-peptide was detectable in 53.8%, 50% and 62.2% participants with diabetes duration ≤ 10 years, 10–20 years and > 20 years respectively (trend *p* = 0.22). In people with childhood-onset diabetes C-peptide was more often detectable in those with longer versus shorter diabetes duration (60% vs. 39%, *p* for positive trend < 0.05). In those with diabetes diagnosed at age ≥ 18y.o there was a non-significant negative trend for the percentage of people with detectable C-peptide and diabetes duration (from 88 to 72%, *p* = 0.07) (Fig. 1B). For those with detectable C-peptide, C-peptide levels did not differ significantly by diabetes duration. There was no difference in detectable C-peptide rates with diabetes duration in participants diagnosed below and above 13 years of age, a common age of diabetes onset and puberty^15^ (ESM Fig. 1). There were no significant differences between C-peptide detectability or levels by diabetes complications status (data not shown).

### C-peptide micro-secretion is associated with lower HbA1c

There were no significant differences in concurrent HbA1c levels by diabetes duration if C-peptide status was not considered. However, participants with diabetes, with detectable versus undetectable C-peptide, had lower concurrent HbA1c levels: 8.2 ± 1.7% (66 ± 18 mmol/mol) versus 8.5 ± 1.4% (70 ± 16 mmol/mol), *p* = 0.001. In those with earlier diabetes onset (< 18 y.o.) HbA1c was lower with detectable versus undetectable C-peptide: 8.4 ± 1.8% (68 ± 19 mmol/mol) versus 8.6 ± 1.5% (70 ± 16 mmol/mol) respectively, *p* = 0.045. Similar results were observed in participants with adult-onset diabetes and detectable versus undetectable C-peptide: (7.7 ± 1.3% (61 ± 15 mmol/mol) versus 8.2 ± 1.1% (66 ± 12 mmol/mol) respectively, *p* = 0.053.

Overall HbA1c levels were significantly lower in participants diagnosed at adult versus childhood age without considering C-peptide detectability status: 7.8 ± 1.3% (62 ± 14 mmol/mol) versus 8.5 ± 1.6% (69 ± 18 mmol/mol), *p* = 0.001. HbA1c was lower in participants with detectable versus undetectable C-peptide in most diabetes duration categories, but reached statistical significance only in participants with 10–20 years diabetes: 7.9 ± 1.3% (64 ± 14 mmol/mol) versus 8.8 ± 1.4% (73 ± 16 mmol/mol), *p* = 0.003.

Detectable C-peptide was not a significant predictor (*p* = 0.06) of concurrent HbA1c reaching recommended targets (< 7% (53 mmol/mol) for adults, < 7.5% (58 mmol/mol) for youth) in models containing age, sex and age of T1D diagnosis (Table 2). Participants with detectable C-peptide had 97% (95%CI 23%, 217%; *p* = 0.005) higher odds of having a HbA1c level < 8% (64 mmol/mol) than those with non-detectable C-peptide in logistic regression (after adjustment for age, sex and age at diabetes onset) (Table 2).

**Table 2:** Predictors of HbA1c target level (logistic regression) and of HbA1c < 8% level (logistic regression).

Model	Standardized coefficient	OR	− 95% CI	+95% CI	*p*
**Predictors of HbA1c target level**					
HbA1c target (*p* = 0.03), Nagelkerke R^2^ = 0.10					
Gender (men)	0.34	1.98	1.15	3.40	**0.01**
Age	0.15	1.01	0.99	1.03	0.37
Age of diabetes onset	− 0.10	0.99	0.96	1.02	0.56
Detectable C-peptide	0.28	1.74	0.97	3.13	0.06
**Predictors of HbA1c < 8% level**					
HbA1c < 8% (*p* = 0.0005), Nagelkerke R^2^ = 0.18					
Gender (men)	0.24	1.64	1.05	2.56	**0.03**
Age	0.25	1.02	1.00	1.03	0.09
Age of diabetes onset	− 0.03	1.00	0.97	1.02	0.83
Detectable C-peptide	0.34	1.97	1.23	3.17	**0.005**

Data shown: Odds ratio (OR), 95% confidence intervals (CI) and *p*-value (p<0.05 in bold).

### miRs profiles in participants with and without diabetes

The microRNA substudy included 305 participants with diabetes and 211 CON. Clinical characteristics are shown in Table 3. Expression of five miRs differed by diabetes status. miR-186 and miR-223 expression were significantly increased while miR-9, miR-22 and miR-125b were significantly decreased in people with diabetes versus CON (ESM Fig. 2 Panel A).

**Table 3:** Clinical characteristics of participants with and without diabetes in the miRs substudy.

	Controls	All Type 1 diabetes	Age at diabetes diagnosis (years)		Diabetes duration (years)	
			< 18	≥ 18	< 16	≥ 16
n (% men)	211 (46)	305 (44)	219 (45)	86 (47)	189 (44)	116 (46)
Age (years)	35 ± 16	30 ± 16*	24 ± 13	45 ± 13**	22 ± 11	43 ± 14***
Type 1 diabetes duration (years)	–	15 ± 12	15 ± 12	15 ± 11	8 ± 4	27 ± 10***
HbA1c (%)	5.1 ± 0.4	8.4 ± 1.6*	8.5 ± 1.6	7.9 ± 1.3	8.5 ± 1.7	8.1 ± 1.4***
HbA1c (mmol/mol)	32 ± 4	68 ± 17*	70 ± 18	63 ± 14**	70 ± 18	65 ± 15
At HbA1c target (%)^a^	–	20	21	18	19	21
Total Cholesterol (mmol/L)	5.1 ± 1.1	4.6 ± 1.0*	4.5 ± 1.0	4.8 ± 0.9**	4.6 ± 1.0	4.7 ± 1.0
HDL –C (mmol/L)	1.5 ± 0.4	1.5 ± 0.4	1.4 ± 0.4	1.6 ± 0.4**	1.4 ± 0.3	1.6 ± 0.5***
LDL-C (mmol/L)	3.1 ± 1.0	2.7 ± 0.9*	2.7 ± 0.9	2.7 ± 0.8	2.7 ± 0.8	2.6 ± 0.9
BMI (kg/m2)	25.5 ± 4.5	25.2 ± 5.0	24.8 ± 5.2	26.4 ± 4.1**	24.2 ± 4.2	26.9 ± 5.7
SBP (mmHg)	121 ± 15	119 ± 17	116 ± 17	126 ± 18**	112 ± 13	131 ± 18***
DBP (mm Hg)	69 ± 10	67 ± 10*	65 ± 10	70 ± 9**	64 ± 9	72 ± 9***
Pulse Pressure (mmHg)	52 ± 9	57 ± 11*	57 ± 11	56 ± 13	52 ± 7	59 ± 13***
Serum creatinine (µmol/L)	80 (70, 84.5)	80 (70, 90)	80 (70, 90)	80 (70, 90)	78 (66, 84)	90 (73, 100)***
ACR (mg/mmol)	0.48 (0.32, 0.80)	0.73 (0.40, 1.60)*	0.75 (0.46, 1.69)	0.68 (0.35, 1.60)	0.69 (0.40, 1.30)	0.90 (0.45, 2.63)***
eGFR (ml/min/m^2^)	106 (92, 120)	104 (81, 131)	108 (82, 134)	100 (84, 124)	123 (96, 139)	97 (72, 117)***
Complications n (%)	–	103 (34)	83 (38)	18 (23)**	49 (26)	51 (47)***
Retinopathy n (%)	–	69 (23)	51 (23)	16 (20)	24 (13)	43 (39)***
Nephropathy n (%)	–	72 (24)	59 (27)	15 (19)	35 (18)	39 (36)***
CVD n (%)	–	20 (7)	11 (5)	6 (8)	3 (1)	10 (9)***

Data shown: mean ± SD or median (LQ, UQ).

*p < 0.05 versus Controls (CON).

**p < 0.05 versus Age at diabetes diagnosis < 18 years.

***p < 0.05 versus diabetes duration < 16 years.

^a^HbA1c target: ≤ 7% (53 mmol/mol) for adults, ≤ 7.5% (58 mmol/mol) for adolescents.

**Figure 2:**
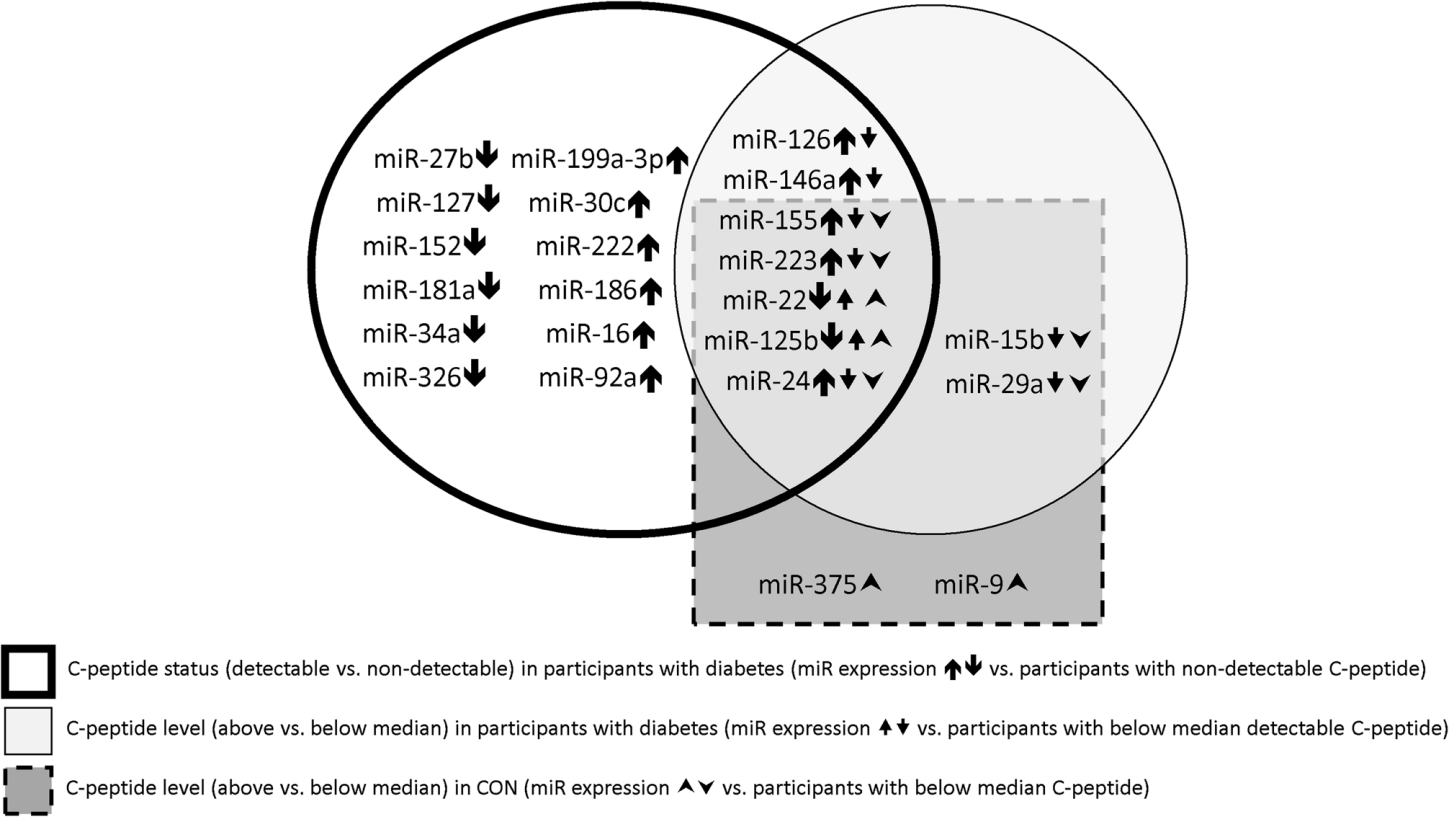
Venn diagram of miRs showing significant expression (Ct) differences in participants with diabetes with versus without detectable C-peptide, participants with and without diabetes with C-peptide levels below and above median. Arrows indicating the miRs abundance difference in comparison to the reference group (: compared with undetectable for C-peptide status, and : compared with below median C-peptide levels in participants with and without diabetes respectively).

### Plasma miRs profiles by age of diabetes diagnosis and diabetes duration

Based on optimal cut-offs, determined with the classification tree using the CART algorithm for age of diabetes onset (DX) (17.5 y.o.) and diabetes duration (DUR) (16 years), participants were divided into four groups:

**Table:** 

	T1D duration < 16 years	T1D duration ≥ 16 years
T1D onset < 17.5 y.o	1. Young DX/Short DUR	2. Young DX/Long DUR
T1D onset ≥ 17.5 y.o	3. Old DX/Short DUR	4. Old DX/Long DUR

Comparing Group 1 and 2 (effect of diabetes duration) 13 miRs were significantly increased in Group 1 and five miRs were significantly increased in Group 2. Comparing Groups 1 and 3 (effect of age of diabetes onset) 14 miRs were significantly increased in Group 1 and one miR in Group 3. Comparing Groups 1 and 4 (combined effect of age of diabetes onset and diabetes duration) 18 miRs were significantly increased in Group 1 versus three miRs in Group 4. There were no significant differences between other groups (Groups 2 vs. 3 or 4 and Groups 3 vs. 4). Twenty-four miRs showed significant differences in all comparisons. Thirteen miRs were significantly different in any of Groups 1 versus 2, 1 versus 3 and 1 versus 4, two miRs were different only in comparison of Groups 1 versus 3 and 1 versus 4, three miRs in 1 versus 2 and 1 versus 4 only and five miRs were significantly different only in one of the comparisons (1 vs. 2 or 1 vs. 4) (ESM Fig. 2 Panels B-D).

### Plasma miRs and C-peptide levels

There were 16 miRs for which their cycle thresholds (Ct) correlated significantly with C-peptide levels in participants with and without diabetes (with detectable C-peptide in 168 participants with diabetes with miRs measured, 52.3% of all participants with diabetes with measured C-peptide and miRs) (ESM Table 1). Nine miRs Ct had statistically significant correlations (all inverse, except miR-375 in the CON group) with C-peptide levels in both groups: people with diabetes with detectable C-peptide and CON (miR-15b, miR-24, miR-126, miR-146a, miR-155, miR-199a-3p, miR-222, miR-223, miR-375).

Exhaustive search analysis for the best linear regression model for detectable C-peptide in CON contained 24 miRs, with an overall adjusted R^2^ = 0.36, *p* < 0.0001. In people with diabetes, the best model contained 15 miRs, with overall adjusted R^2^ = 0.18, *p* < 0.0001 (ESM Table 2). Among eight miRs common between people with and without diabetes (miR-7, miR-125a-5p, miR-125b, miR-127, miR-148a, miR-16, miR-375, miR-93) only three showed the same direction of the regression standardised coefficient (all positive) in both groups (miR-125b, miR-127, miR-16).

In an exhaustive search for a logistic regression for determinants of detectable C-peptide status in participants with diabetes the best model contained 14 miRs with R^2^ = 0.46, *p* < 0.0001 (ESM Table 3).

Penalised logistic regression identified nine microRNAs associated with detectable C-peptide in people with diabetes (miR-22, miR-24, miR-103, miR-152, miR-155, miR-181a, miR-210, miR-223 and miR-374). A ROC curve constructed with these miRs has an AUC of 0.74 and overall accuracy of 72%, *p* < 0.0001 for prediction of detectable C-peptide in people with diabetes (ESM Fig. 3).

Using the Boruta algorithm approach in participants with diabetes 13 miRs were associated with C-peptide status (miR-26a, miR-99b, miR-146a, miR-30b, miR-22, miR-127, miR-125a-5p, miR-223, miR-126, miR-199a-3p, miR-24, miR-155 and miR-181a, ESM Fig. 4) and seven with C-peptide levels (miR-127, miR-99b, miR-126, miR-27b, miR-223, miR-125b, miR-24, ESM Fig. 5). In CON 31 miRs were selected by this algorithm as non-redundant (ESM Fig. 6). ESM Fig. 7 shows the Venn diagram for miRs selected by the Boruta algorithm.

We compared miRs levels (assessed in Ct) between participants (1) with diabetes with and (2) without detectable C-peptide, (3) with detectable C-peptide level below and (4) above the median level (5.02 pmol/L) and between (5) CON subjects with C-peptide level below and (6) above the median level (531.64 pmol/L). Figure 2 is a Venn diagram of miRs with differential expression between CON and participants with diabetes with and without detectable C-peptide. Eight miRs had lower expression and 11 had higher expression in people with diabetes without detectable C-peptide. Two miRs had higher and seven had lower abundance in people with diabetes with detectable C-peptide below versus above the median C-peptide value. Four miRs had higher and five had lower abundance in CON participants with C-peptide levels below the median. Five miRs (miR-155, miR-223, miR-22, miR-125b and miR-24) were common to the three groups.

## Discussion

Using a relatively recently available ultrasensitive C-peptide assay in a cross-sectional study we demonstrated insulin microsecretion (detectable C-peptide levels) in 55.3% of people with diabetes, including 64.1% of adults and 34.0% of youth with diabetes. Those with adult-onset diabetes had detectable C-peptide significantly more often than those diagnosed during youth (79% vs. 43%). Those with childhood-onset diabetes and longer diabetes duration also had detectable C-peptide significantly more often than those with shorter diabetes duration (increasing from 39 to 60%). This merits confirmation in longitudinal studies. In youth with diabetes C-peptide detectability or level did not differ by autoantibody status at 8.4 years post-diagnosis. Of 50 miRs potentially related to beta cell death plasma levels of 5–21 miRs differed significantly by diabetes status (vs. CON), by age of diabetes onset and duration. We also identified correlations between C-peptide and plasma levels of 16 miRs in CON and with 12 miRs in participants with diabetes (with six overlapping). Hence across a wide range of age and diabetes duration we demonstrate high rates of low-level C-peptide, supporting the hypothesis that beta cells may reactivate or regenerate, and that circulating miRs may reflect beta cell status.

A high sensitivity C-peptide assay, with a lower detection limit 40-fold lower than that of most clinical and older research assays was used to analyse plasma from fasted adults and non-fasted youth. We recognise that fasting C-peptide levels may be lower than in the post-prandial state and post-stimulation. In participants with diabetes with undetectable C-peptide a mixed meal test increased C-peptide to detectable post-prandial levels in ≈10% of subjects^8^. Hence our percentage of individuals with detectable C-peptide may be an underestimate.

Residual insulin secretion in diabetes has clinical importance regarding glycaemia. We found that detectable C-peptide was associated with higher rates of HbA1c < 8%, but not with significantly higher rates of achievement of the lower recommended target HbA1c levels. We did not find a correlation between detectable C-peptide and HbA1c levels. In keeping with our results, a study by Oram et al. in 924 people with Type 1 diabetes found no relationship between urinary C-peptide/creatinine ratio, HbA1c or exogenous insulin dose^11^.

We did not find any differences between C-peptide detectability and chronic complications. Others have reported that residual C-peptide is associated with fewer vascular complications^6,7,12^ and with less SH^5,12^. In the Joslin Medallist Study (diabetes duration ≥ 50 years), who were relatively free of chronic complications, C-peptide was present in 67% using less-sensitive C-peptide assays (> 30 pmol/L) than herein. While survival bias is likely, these data support benefit of residual beta cell function^2,7^. Similarly, the DCCT study reported a C-peptide threshold > 200 pmol/L to be protective for chronic complications and SH^6,16^. Kuhtreiber et al. (n = 1272) utilising an ultrasensitive C-peptide assay identified a protective threshold of > 10 pmol/L for complications regardless of diabetes duration^12^.

Autoantibodies against pancreatic cell components are detectable prior to Type 1 diabetes diagnosis and for several years after, though often wane after longer diabetes duration^17–19^. We did not find any relationship between C-peptide and autoantibody status in the youth with diabetes in our cross-sectional study. Other publications have reported mixed data. In two studies of paediatric patients with diabetes at diagnosis and after seven years, ICA512/IA2 persistence was associated with presence of C-peptide, better glycaemia and lower insulin requirement, suggestive of preserved beta cell function^20^. Another study with younger diabetes onset was associated with low C-peptide levels, which was not associated with autoantibody status at diagnosis^20^.

In our cross-sectional study we found differences in C-peptide detectability by age of diabetes diagnosis and diabetes duration. These results are in keeping with and extend existent literature showing that presence of detectable C-peptide is not uncommon in long diabetes duration and that adult-onset diabetes is associated with more rapid C-peptide production loss (e.g. with age at onset 6–10 years C-peptide production was observed for 20 years vs. less than 10 years with onset at age of > 40 years)^10^. Rapid C-peptide level decline with younger diabetes onset and a slower decline in C-peptide levels with increasing diabetes duration (up to 73 years) have been reported by others using routine^3,4,21^ and ultrasensitive C-peptide assays^9–11^. Madsbad et al.reported a higher prevalence of detectable C-peptide in those with older diabetes onset (30–40 y.o.) vs. childhood onset (10–20 y.o.) and documented C-peptide decline in the first two years post-diagnosis in the younger cohort^3^. During DCCT screening, in 13–39 y.o. patients with 1–15 years of diabetes, C-peptide levels were lower with longer duration, and stimulated C-peptide levels were lower in adolescents versus adults at 1–5 years diabetes duration; supporting a sharper early decline in C-peptide in younger patients^4^. In the SEARCH cohort of youth with diabetes of relatively short duration (age range 1–23 years, mean duration three years), clinically significant C-peptide levels were found in adolescents up to five years post-diagnosis, an age group considered to have rapid C-peptide falls. This study also showed that fasting C-peptide was higher at diabetes onset in adolescents compared to younger children, and levels declined between < 1 to 6 + years duration^21^. Using ultrasensitive assays the Type 1 Diabetes Exchange Clinical Network found higher C-peptide in adult versus youth diabetes onset, and decline in residual C-peptide with longer diabetes duration regardless of age of diagnosis^9^. Wang et al. found a significantly higher proportion of detectable C-peptide with shorter diabetes duration (79% at < 5 years vs. 10% at 31–40 years duration) and in adult versus early childhood onset^10^. Unexpectedly there were low C-peptide levels despite short diabetes duration in those diagnosed after age 40 years. In the UNITED Team cohort of people with diabetes diagnosed at < 30 y.o., 80% had detectable urine C-peptide at > 5 years diabetes duration. There was a higher proportion of detectable C-peptide with shorter duration, although in contrast to a previous study, this was not related to age of diagnosis^11^. The differences in these studies using ultrasensitive assays demonstrate heterogeneity of Type 1 diabetes, which may be related to age of diabetes onset and other unmeasured factors.

Our finding, albeit in a cross-sectional study, of an increasing proportion of detectable C-peptide with longer diabetes duration diagnosed at age ≤ 18 years is novel. We also note that the absence of C-peptide is only present in those within the first five years post-diagnosis in the childhood-onset group and the detectable C-peptide levels rates are lower than the adult-onset group at all diabetes durations. Some previous studies have demonstrated a non-significant trend to higher rates of detectable C-peptide after prolonged diabetes > 30 years in those with young age diabetes onset^3,9^. While we cannot draw conclusions from a cross-sectional study, animal data support greater beta cell regeneration capacity with younger diabetes onset. In a TIF-1A mouse model of progressive beta cell decline adaptive proliferation of remaining beta cells was found. Furthermore, precursor cells were less likely to contribute to beta-cell regeneration in adult mice than in younger mice. This study also raised other possibilities which may explain insulin recovery, with beta cells expressing glucagon; suggesting transition between alpha and beta cell identities^22^. Earlier human islet cell morphology studies have also shown evidence of beta cell regeneration or neotransformation in patients with diabetes of varying duration^6^. It was recognised that islet cells in diabetes were not atrophic, as previously thought, but “pseudo-atrophic” containing glucagon- and somatostatin-containing cells, in addition to atypical regeneration with pancreatic-polypeptide-containing cells^23^. There were also various forms of regeneration, including new hyperactive beta cells from proliferation of excretory duct epithelial cells and islet cell hyperplasia with large beta cells containing immunoreactive insulin in “pseudo-atrophic” pancreas^23^.

Another novel aspect to our cross-sectional study is that we explored associations between C-peptide and circulating miRs from a signature of pancreatic beta-cell death^14^. We identified eight miRs showing the same direction and strength of correlation with C-peptide levels in people with and without diabetes. Not surprisingly, not all miRs selected in exhaustive search analyses were associated with C-peptide status in penalised logistic regression analyses as the latter selects only one variable from a group of correlated factors. miRNAs with the strongest association with detectable C-peptide level were miR-181a and miR-155. miRNA-181a has been previously described as dysregulated in people with Type 1 diabetes^24,25^. miRNA-155 has shown an opposite direction of the association (with lower miR expression C-peptide detectability was increasing). miR-155 has also been associated with autoimmunity and inflammatory status in people with Type 1 diabetes^26^. miRNA-199a-3p levels are known to increase in circulation prior to hyperglycaemia^27^. In our study increased miR-199a-3p Ct (i.e. decreased expression) was associated with lower C-peptide levels. miRNA-24 may have roles in adiposity and insulin resistance in Type 2 diabetes^28^. We have summarised our findings related to miRs differentially expressed in people with and without Type 1 diabetes and in some of these patients with detectable versus non-detectable C-peptide against some literature in ESM Table 4^29–34^. Relatively few miRs were associated with C-peptide levels in Type 1 diabetes versus over 20 miRs in the group of individuals without diabetes. miRs have functional effects and can be both a therapeutic target or agent. Our results support that the evaluation of miRs in humans at risk of or with Type 1 diabetes may be a useful adjunct to genetic and autoantibody studies in the early diagnosis of pre-Type 1 diabetes and its monitoring and treatment, particularly given that genetics and autoantibodies may differ by ethnicity, yet beta cell loss is common to Type 1 diabetes^17–19^. Cultured islet, cell and animal studies relating miRs and C-peptide production are also merited.

Study strengths include analyses of adults and paediatric samples with wide ranges of diabetes onset and duration, use of an ultrasensitive C-peptide assay and inclusion of novel miRs markers of beta cell death. Study limitations include the cross-sectional nature, lack of SH data, and that fasting C-peptide was measured in adults and non-fasting levels were measured in youth. Non-fasting status would tend to increase C-peptide. Selection bias may exist as all subjects with Type 1 diabetes were attending tertiary referral hospital diabetes clinics, as is recommended in Australia. In the miR C-peptide studies overfitting of statistical models may be a risk, but the aim was not to find formulae for C-peptide levels but to find non-redundant miRs associated with C-peptide status and level. We acknowledge that non-studied miRs may also be relevant and some important miRs may be below our assay limit of detection. Cellular miRs which may be at higher levels are of interest.

In conclusion, our results support (a) greater loss of beta cell function in younger diabetes onset; (b) persistent insulin secretion, particularly with later diabetes onset and longer duration; and (c) relationships of C-peptide levels with circulating miRs. Confirmatory studies, including longitudinal studies are merited. Identifying a sub-population of patients with diabetes with greater residual beta cell function or potential for beta cell recovery or regeneration may enhance success of beta-cell targeted clinical trials. Some of the miRs identified may also be of use in the early diagnosis and monitoring, including of therapies, in people with pre-Type 1 diabetes and recent onset Type 1 diabetes.

## Methods

The study was approved by St Vincent’s Hospital Melbourne (SVHM) and the Children’s Hospital at Westmead (CHW) Human Research Ethics Committees. Each participant, or for minors their legal guardian, provided written informed consent. All research methods were performed in accordance with the relevant guidelines.

### Participants

Participants with diabetes (n = 349, 249 adults) were recruited from adult (SVHM) and paediatric (CHW) diabetes clinics, which are tertiary referral clinics. Adult participants without diabetes (CON) (n = 220) were recruited from a research volunteer registry. Paediatric CON participants (n = 33) were recruited from siblings without diabetes and/or visitors to the hospital. Adults were ≥ 18 years of age. Arbitrary pre-stated diabetes duration cut-points were ≤ 10 years, 10–20 years and > 20 years. HbA1c thresholds analysed were clinically recommended target ranges (< 7%, 53 mmol/mol) for adults and (< 7.5%, 58 mmol/mol) for the paediatric age group, and < 8% (64 mmol/mol) reflecting the mean HbA1c for Australians with diabetes^35^.

A history and clinical examination were performed. Height (by stadiometer) and weight (by digital scale) were used to calculate BMI. Obesity was defined as per International Obesity Task Force Definitions^36^. Blood pressure was taken as the mean of three blood pressure readings in a supine subject after at least 5-min rest. Venous blood was collected from fasted adults, and generally non-fasting youth.

The microRNA substudy included all study participants with adequate residual plasma (n = 516, including 386 adults (206 with diabetes) and 130 youth (99 with diabetes)).

### T1D complications

Paediatric and adult participants were classified as being free from microvascular and/or macrovascular complications (CX-) or having such complications (CX +). CX + included ≥ 1 of diabetic retinopathy (DR)^37^ and/or diabetic nephropathy (DN)^38^, with age-based definitions^39,40^. Of the 349 participants with diabetes, 221 were CX- and 117 CX + . Complication status was unknown in 11 participants. Only 21 adults had macrovascular complications (cardiovascular disease (AMI, angina, positive Rose questionnaire), transient ischaemic attack (TIA), stroke or vascular bypass procedure), all of whom also had microvascular complications.

In youth DR was defined as ≥ 1 microaneurysm/haemorrhage in either eye as per the early treatment of Diabetic Retinopathy Study (ETDRS) adaptation of the modified Airlie House classification (level 21, non-proliferative DR (NPDR) or greater)^41^. In adults DR was defined as clinically significant proliferative DR (PDR) or pre-PDR requiring laser treatment. (Intraocular injections were not widely used at the time of study conduct, which was prior to 2014).

In youth DN was defined as an albumin excretion rate (AER) > 7.5 μg/min from three overnight, timed urine collections. This cut-off is > 95th percentile of normal adolescents and predicts albuminuria^42,43^. In adults increased AER was > 15ug/min in ≥ 2 of three 12 or 24-h urine collections.

### C-peptide measurement

Plasma (EDTA) was stored (-80ºC) until analysis by ultrasensitive ELISA (Mercodia, Sweden) with a detection limit of 1.25 pmol/L (0.0038 ng/mL) as per the manufacturer’s instructions. Undetectable levels were expressed as ¼ of the assay’s lowest calibration as per manufacturer’s instructions (Tech. note 34–0144). Intra and inter-assay coefficients of variation were 3.2% and 5.8% respectively. C-peptide levels from CON were quantified in the same ELISA at 20-fold dilution, with the analyst masked to sample identity. Each assay plate contained samples from individuals with and without diabetes.

### Autoantibody status in paediatric participants with diabetes

Tyrosine phosphatase-related islet antigen 2 (IA2) and glutamic acid decarboxylase 65 (GAD) titres were measured by radioimmunoassay as per manufacturer’s instructions (RSR Ltd, Cardiff, United Kingdom) at diabetes diagnosis or at a complications screening visit. Results analysed were from the time-point immediately preceding C-peptide sample collection for this project.

### RNA extraction and miR analysis

Briefly, glycogen was added to the sample (100µL plasma) followed by the addition of 500µL Trizol (Thermo Scientific). A synthetic spike-in control Arabidopsis thaliana miRNA (ath-miR-172a) was added for correcting any loss during RNA isolation step. Chloroform was used to separate RNA-containing aqueous phase after centrifugation. Subsequent isolation steps were performed on an automated QIAcube HT system using RNeasy 96 QIAcube HT Kit (Qiagen). The system was used to collect the aqueous phase into new tubes, precipitate, wash and elute RNA. Concentration was measured (Eon, Biotek, Winooski, VT) before setting up the reverse transcription (RT). A custom miR-chip was generated based on a miRNAs signature available through the patent application WO2019000015A1^14^. Reverse transcription was performed with a starting RNA amount of 10 ng using miRNA RT kit (Thermo Scientific) and custom RT primer pool. A synthetic spike-in control Arabidopsis thaliana miRNA (ath-miR-159a) was added to RT. Pre-amplification was carried out before setting up the real-time PCR on an OpenArray platform^44,45^. Data for miRNA expression were normalised to spike-in controls and then analysed.

### Statistics

Statistical analyses used Statistica for Windows ver. 13 (Tibco Software, Palo Alto, CA), XLStat (AddinSoft, Paris, France) and R ver. 3.6 (R Core Team (2019), R Foundation for Statistical Computing, Vienna, Austria). C-peptide levels were log-transformed. Associations between the frequency of C-peptide detectability in the diabetes duration categories were assessed using the Cochran-Armitage test. For the microRNA sub-study, optimal cut-offs for age of diabetes diagnosis and duration were determined with a classification tree using a CART algorithm and Gini impurity measure. For exhaustive search analyses, model selection was performed based on maximised adjusted R^2^ value (linear regression) or minimised AIC value (logistic regression). Variables importance was tested using a Random Forest method with the Boruta algorithm^46^. C-peptide, HbA1c and miRs results between groups were compared using parametric and non-parametric methods as appropriate. Associations between variables were assess using the Spearman correlation, segmental linear and logistic regression. Bonferroni correction for multiple comparisons was used where applicable. Significance was taken at *p* < 0.05.

## Supplementary information

Supplementary Information.
