# Supplementary material for: Insulin micro-secretion in Type 1 diabetes and related microRNA profiles
Source: Sci Rep. 2021 Jun 3;11:11727. doi: 10.1038/s41598-021-90856-6 (PMC8175359; doi:10.1038/s41598-021-90856-6)

**Electronic supplementary materials**

Januszewski AS†*^1,2^, Cho YH†^3,4^, Joglekar MV†^1,5^, Farr R^1^, Scott E^1^, Wong WKM^1,5^, Carroll LM^1^, Loh YW^1^, Benitez-Aguirre PZ^3,4^, Keech AC^1^, O’Neal DN^2^, Craig ME^3,4^, Hardikar AA‡^1,5,6^, Donaghue KC‡^3,4^, Jenkins AJ‡*^1,2^

**Insulin micro-secretion in Type 1 diabetes and related microRNA profiles**

† - equal First Authors

‡ - equal Senior Authors

**Affiliations**

^1^NHMRC Clinical Trials Centre, University of Sydney, Sydney, Australia

^2^Department of Medicine, University of Melbourne, Melbourne, Australia

^3^Discipline of Paediatrics and Child Health, University of Sydney, Sydney, Australia

^4^Institute of Endocrinology and Diabetes, The Children’s Hospital at Westmead, Sydney, Australia

^5^School of Medicine, Western Sydney University, Sydney, Australia

^6^Department of Science and Environment, Roskilde University, Copenhagen, Denmark

ESM Table 1

Spearman correlation coefficients of associations between miRs and C-peptide levels in CON and participants with diabetes (with detectable C-peptide).

|  | CON | | Diabetes | |
| --- | --- | --- | --- | --- |
|  | R | p | R | p |
| miR-9 | 0.20 | **0.004** |  |  |
| miR-15b | -0.15 | **0.03** | -0.17 | **0.03** |
| miR-22 | 0.25 | **0.0002** |  |  |
| miR-24 | -0.29 | **<0.0001** | -0.21 | **0.005** |
| miR-29a | -0.25 | **0.0003** |  |  |
| miR-125a-5p | -0.14 | **0.046** |  |  |
| miR-125b | 0.23 | **0.0007** |  |  |
| miR-126 | -0.25 | **0.0002** | -0.17 | **0.03** |
| miR-127 | 0.14 | **0.047** |  |  |
| miR-146a | -0.22 | **0.002** | -0.15 | **0.049** |
| miR-155 | -0.25 | **0.0002** | -0.16 | **0.04** |
| miR-186 | -0.18 | **0.007** |  |  |
| miR-199a-3p | -0.15 | **0.03** | -0.17 | **0.03** |
| miR-222 | -0.17 | **0.01** | -0.15 | **0.049** |
| miR-223 | -0.26 | **0.0001** | -0.20 | **0.01** |
| miR-375 | 0.17 | **0.01** | -0.18 | **0.02** |

ESM Table 2

Multiple regression coefficients for best linear model of determinants for C-peptide level in CON and participants with diabetes with detectable C-peptide.

|  | CON |  | Diabetes |  |
| --- | --- | --- | --- | --- |
| miR | Standardized coefficient | p-value | Standardized coefficient | p-value |
| miR-7 | -0.12 | 0.05 | 0.12 | 0.14 |
| miR-125a-5p | -0.16 | **0.04** | 0.32 | **0.005** |
| miR-125b | 0.11 | 0.13 | 0.26 | **0.02** |
| miR-126 | -0.68 | **0.003** |  |  |
| miR-127 | 0.21 | **0.02** | 0.25 | **0.02** |
| miR-145 | 0.10 | 0.23 |  |  |
| miR-146a |  |  | 0.46 | 0.15 |
| miR-148a | -0.18 | **0.01** | 0.16 | 0.07 |
| miR-152 | -0.10 | 0.19 |  |  |
| miR-16 | 0.24 | 0.31 | 0.42 | 0.14 |
| miR-199a-3p |  |  | -0.30 | **0.01** |
| miR-200a |  |  | -0.08 | 0.28 |
| miR-20a | 0.20 | 0.18 |  |  |
| miR-210 |  |  | -0.19 | 0.05 |
| miR-22* | 0.29 | **0.02** |  |  |
| miR-223 | -0.23 | 0.05 |  |  |
| miR-24 | -0.25 | 0.13 |  |  |
| miR-26a | 0.20 | 0.12 |  |  |
| miR-27a |  |  | -0.24 | 0.07 |
| miR-27b | -0.22 | **0.01** |  |  |
| miR-29a | -0.16 | 0.11 |  |  |
| miR-301b | 0.07 | 0.28 |  |  |
| miR-30a-5p | -0.30 | 0.05 |  |  |
| miR-30e-3p | -0.08 | 0.30 |  |  |
| miR-326 |  |  | -0.14 | 0.17 |
| miR-340* | 0.13 | **0.04** |  |  |
| miR-374 | 0.15 | 0.08 |  |  |
| miR-375 | 0.15 | **0.03** | -0.25 | **0.008** |
| miR-9 |  |  | -0.08 | 0.26 |
| miR-92a | 0.36 | 0.15 |  |  |
| miR-93 | 0.28 | 0.17 | -0.76 | **0.01** |

ESM Table 3

Predictors of detectable C-peptide status (yes/no) in participants with diabetes (logistic regression). Data shown: Odds ratio (OR), -95%/+95% confidence interval (CI) and p-value.

| Model | Standardized coefficient | OR | -95% CI | +95% CI | p |
| --- | --- | --- | --- | --- | --- |
| Detectable C-peptide (p<0.0001, Negelkerke R^2^=0.46) | | | | | |
| miR-24 | 0.30 | 1.22 | 1.02 | 1.45 | **0.03** |
| miR-29a | -0.39 | 0.94 | 0.87 | 1.01 | 0.08 |
| miR-146a | -0.88 | 0.87 | 0.73 | 1.04 | 0.12 |
| miR-155 | 0.44 | 1.06 | 1.01 | 1.11 | **0.02** |
| miR-199a-3p | 0.46 | 1.07 | 0.99 | 1.16 | 0.09 |
| miR-222 | -0.20 | 0.97 | 0.91 | 1.04 | 0.44 |
| miR-374 | -0.27 | 0.96 | 0.91 | 1.01 | 0.08 |
| miR-223 | 0.44 | 1.05 | 0.98 | 1.13 | 0.16 |
| miR-409-5p | 0.30 | 1.23 | 1.01 | 1.51 | **0.04** |
| miR-625 | 0.20 | 1.07 | 0.97 | 1.18 | 0.19 |
| miR-103 | -0.45 | 0.93 | 0.89 | 0.98 | **0.008** |
| miR-7 | -0.23 | 0.90 | 0.77 | 1.04 | 0.14 |
| miR-210 | -0.29 | 0.96 | 0.92 | 1.00 | 0.06 |
| miR-181a | -0.27 | 0.96 | 0.91 | 1.01 | 0.09 |

ESM Table 4

miRs with different expression in subject with vs. without detectable C-peptide (see Fig. 2) and its levels/function reported in Type 1 diabetes.

| miR | Expression in people with diabetes | Expression in people with diabetes with detectable C-peptide vs people with non-detectable C-peptide (this manuscript) |
| --- | --- | --- |
| miR-016 | Lower in diabetes vs. CON [24] | Higher |
| miR-022 | Lower in diabetes vs. CON [24] | Lower |
| miR-024 | Lower in diabetes vs. CON [24,25] | Higher |
| miR-027b | Higher in diabetes vs. CON [25] | Lower |
| miR-030c | Higher in diabetes protects against diabetic cardiomyopathy [34] | Higher |
| miR-034a | Higher in diabetes vs. CON [24] | Lower |
| miR-092a | Lower in diabetes increasing glucose-induced insulin secretion [32] | Higher |
| miR-125b | Higher in diabetes vs. CON [24] | Lower |
| miR-126 | Higher in diabeets enhances cardiac repair after injury [33] | Higher |
| miR-127 | Downregulated during differentiation into insulin-producing cells [31] | Lower |
| miR-146a | Lower in diabetes vs. CON [26] | Higher |
| miR-152 | Higher in diabetes vs. CON [25] | Lower |
| miR-155 | Higher in diabetes vs. CON [26] | Higher |
| miR-181a | Higher in diabetes vs. CON [24,25] | Lower |
| miR-186 | Lower muscle expression in diabetes [30] | Higher |
| miR-199a-3p | Higher in diabetes vs. CON [27] | Higher |
| miR-222 | Lower in diabetes vs. CON [24] | Higher |
| miR-223 | Lower in diabetic nephropathy [29] | Higher |
| miR-326 | Lower in diabetes vs. CON [26] | Lower |

ESM Figure 1.

Comparison of proportion of detectable C-peptide in participants with diabetes stratified by age of diabetes diagnosis (cut-off 13 yrs) and duration.


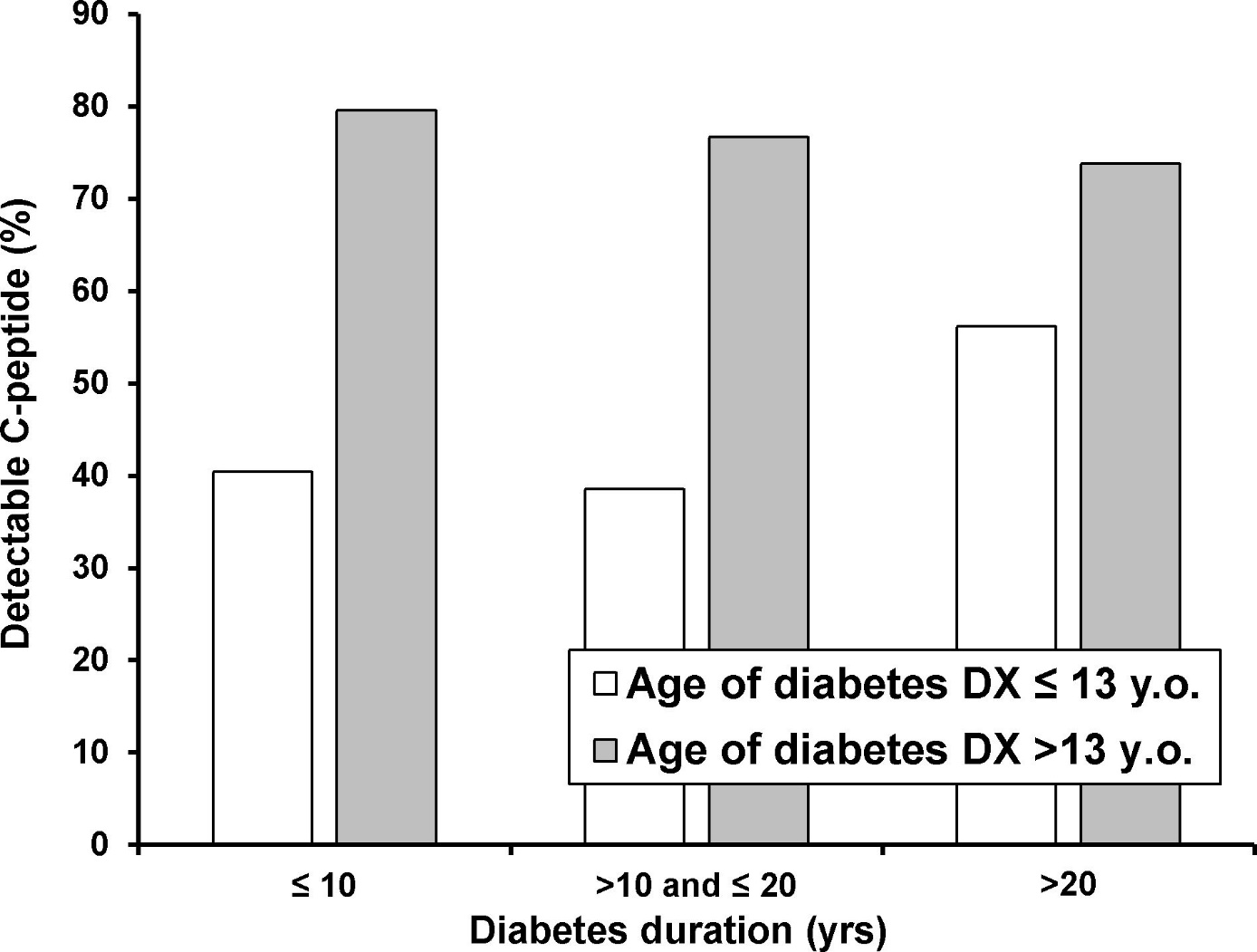


ESM Figure 2.

Panel A. Volcano plot of miRs expression (Ct) in participants with and without diabetes. The horizontal line indicates p=0.05 after Bonferroni correction and vertical lines indicate a one cycle threshold (Ct) difference. Comparison using parametric tests.

Panels B-D. Volcano plots of miRs expression (Ct) in between participants with diabetes with young/old diabetes diagnosis and short/long diabetes duration. Horizontal line indicating p=0.05 after Bonferroni correction and vertical lines indicating one Ct (i.e. two-fold) difference. Comparisons using parametric test.

ESM Figure 3.

ROC curve of C-peptide detectability status using miRs identified by penalized logistic regression as significant determinants of C-peptide status.


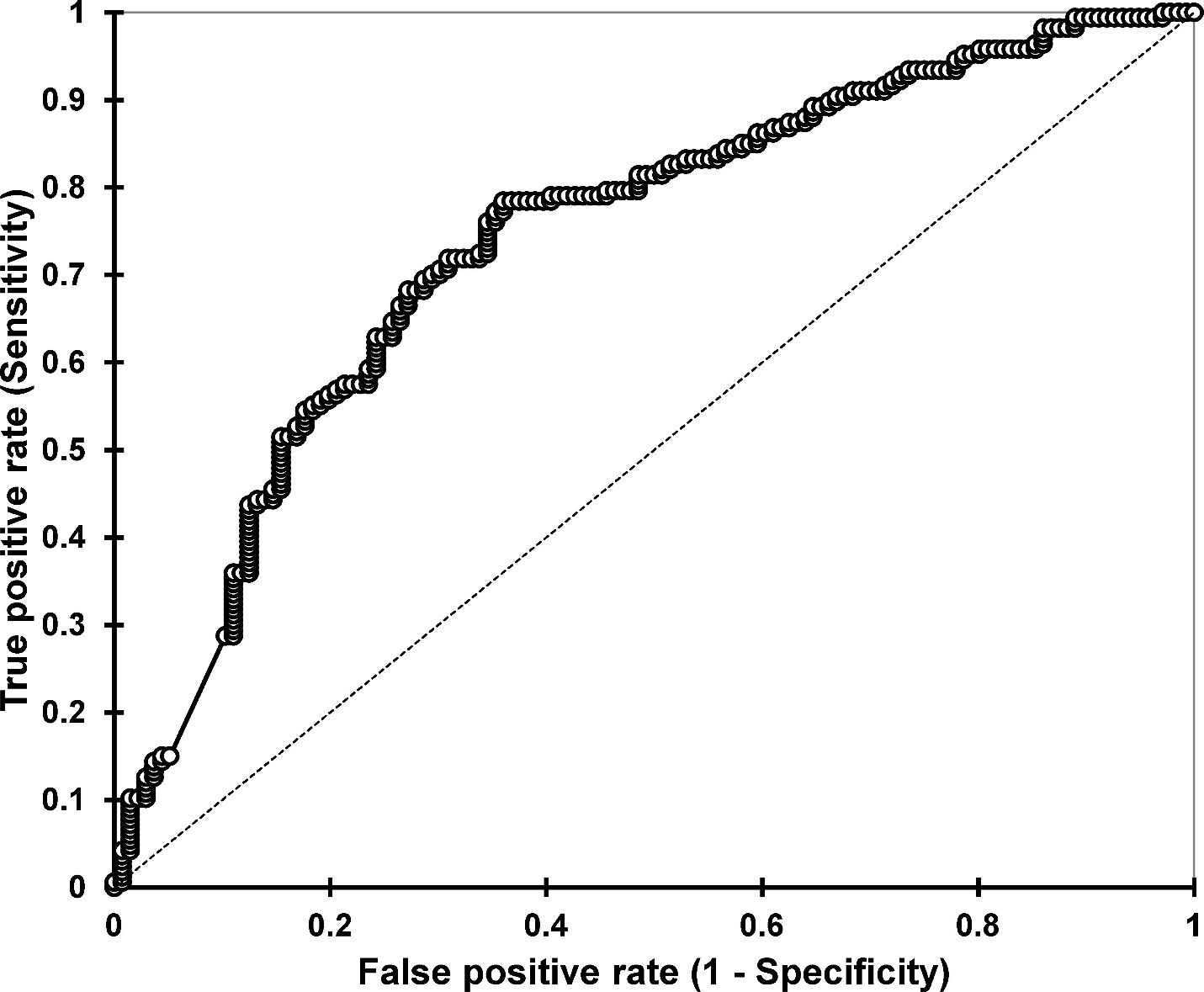


ESM Figure 4.

MiRs associated with presence of C-peptide (detectable level yes/no) in people with diabetes. Importance (difference between Z-score of a feature and a maximum Z-score of its shadow feature) represent the relative importance of miRs in the determination of detectable C-peptide presence in diabetes. White and grey boxplots represent confirmed and rejected features, respectively. Black boxplots represent minimum, average and maximum Z-score of a shadow feature.


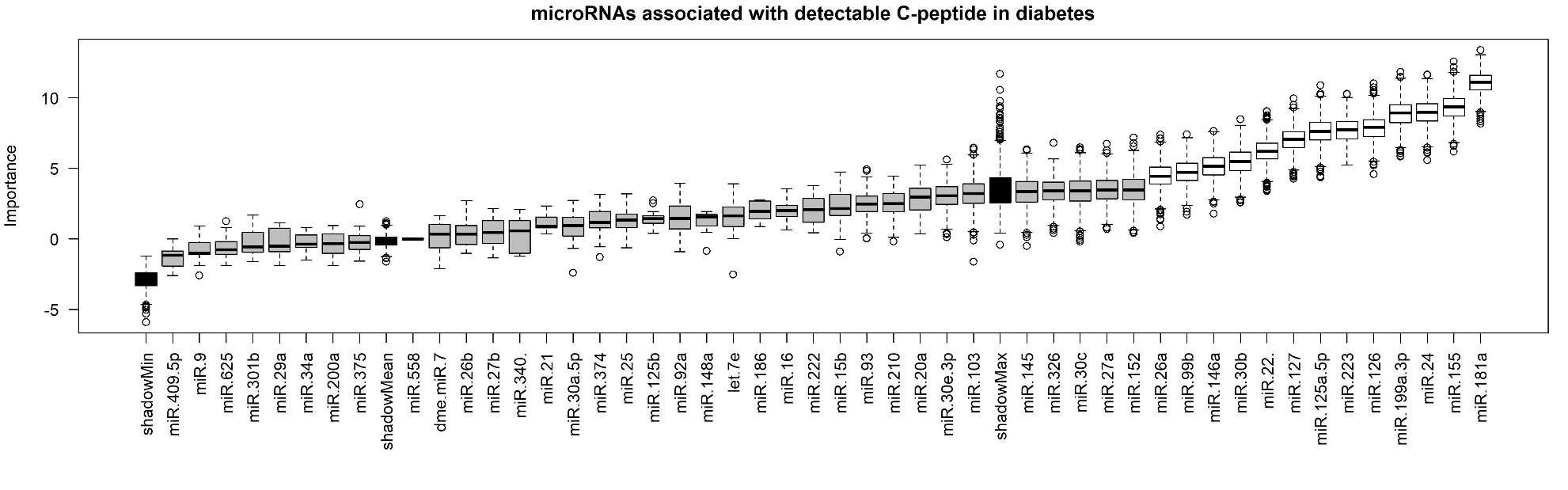


ESM Figure 5.

MiRs associated with presence of C-peptide (detectable level yes/no) in participants with diabetes. Figure setup as ESM Fig 4.


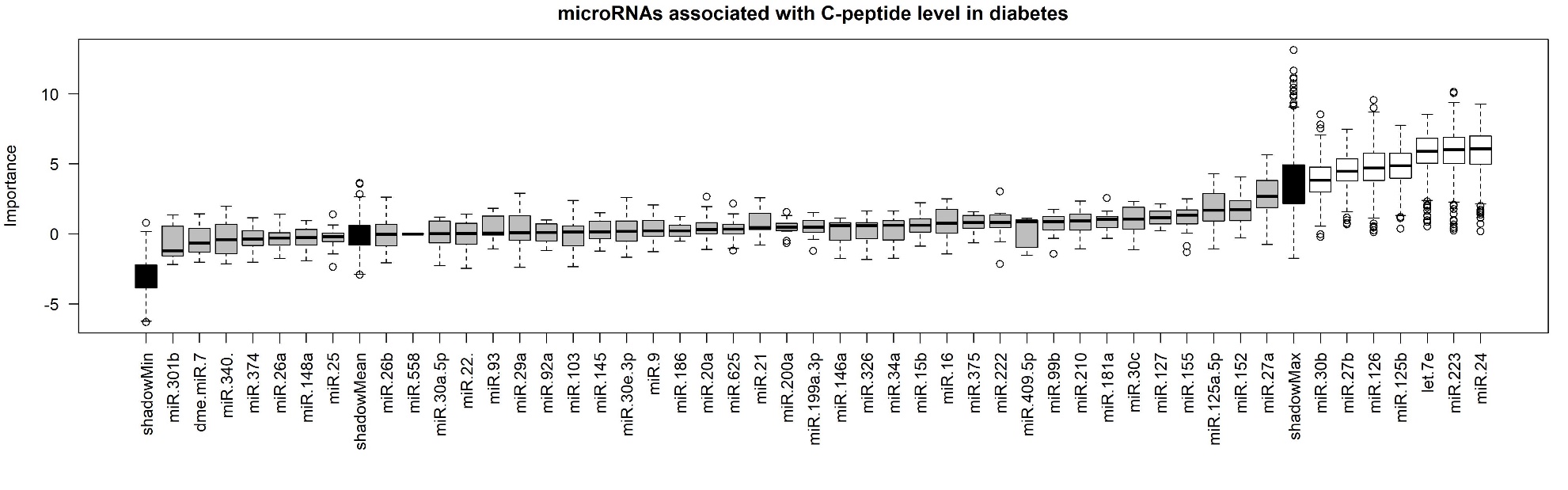


ESM Figure 6.

MiRs associated with C-peptide level in CON. Figure setup as ESM Fig 4.


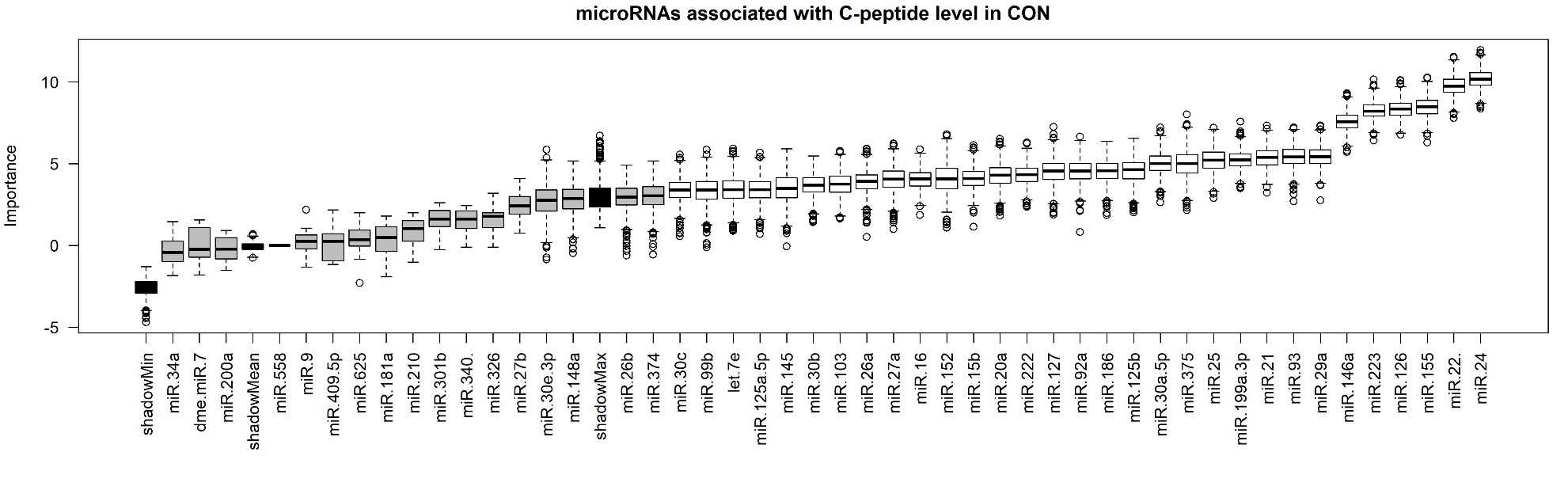


ESM Figure 7.

Venn diagram of miRs selected by the Boruta algorithm as non-redundant for determination of C-peptide status and level in people with and without diabetes.


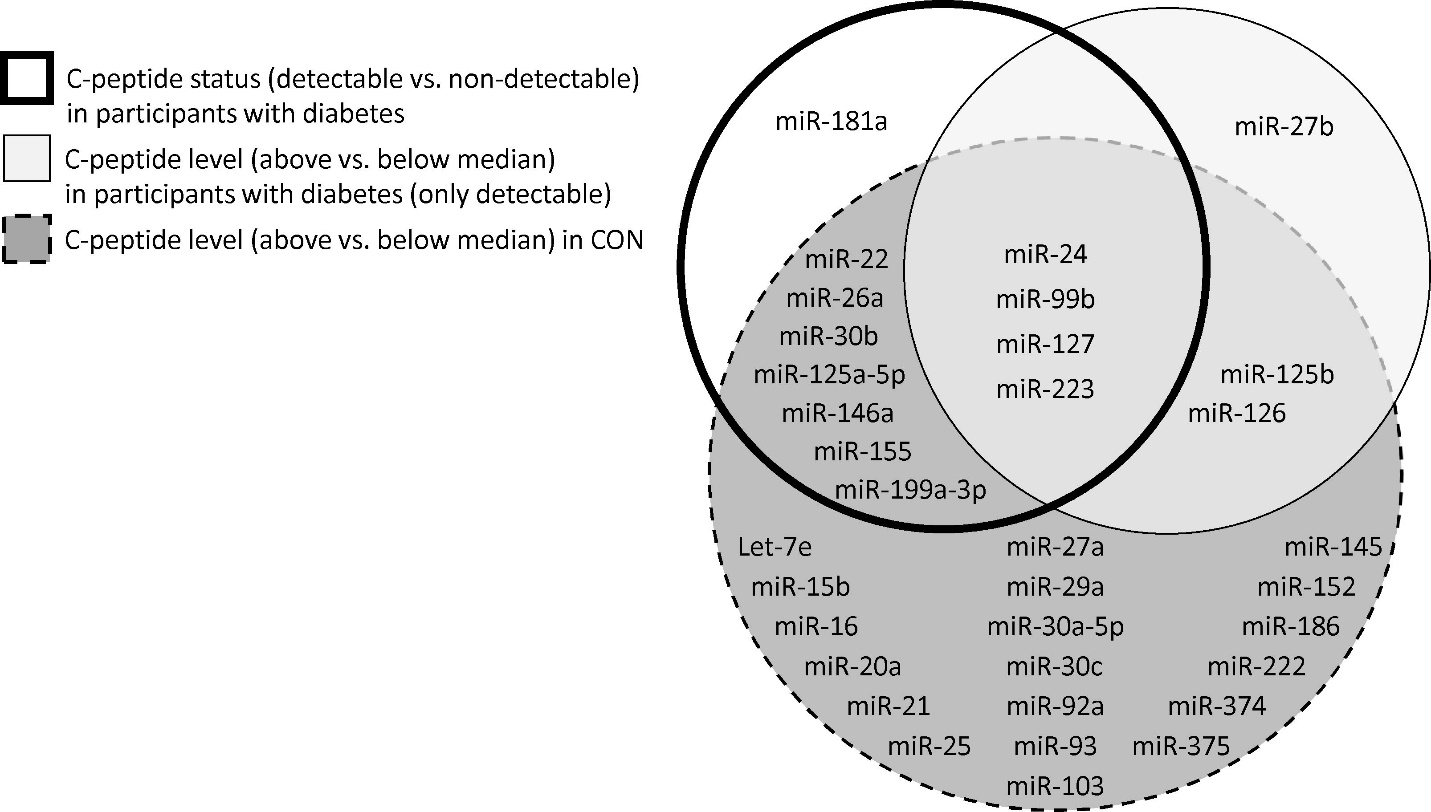

Supplement: Supplementary file 1 — Supplementary Information. [file 41598_2021_90856_MOESM1_ESM.docx]
